# Active phase prebiotic feeding alters gut microbiota, induces weight-independent alleviation of hepatic steatosis and serum cholesterol in high-fat diet-fed mice

**DOI:** 10.1016/j.csbj.2020.12.011

**Published:** 2020-12-24

**Authors:** Shreya Ghosh, Xin Yang, Linghua Wang, Chenhong Zhang, Liping Zhao

**Affiliations:** a State Key Laboratory of Microbial Metabolism, Joint International Research Laboratory of Metabolic & Developmental Sciences, School of Life Sciences and Biotechnology, Shanghai Jiao Tong University, Shanghai 200240, China; b Department of Biochemistry and Microbiology and New Jersey Institute for Food, Nutrition and Health, School of Environmental and Biological Sciences, Rutgers University, New Brunswick, NJ 08901, USA

**Keywords:** NAFLD, Short-chain fatty-acids, Western diet, Soluble fiber, Microbiota modulation, Timed feeding of prebiotics, Cholesterol-lowering effect

## Abstract

Growing evidence suggests that prebiotics may induce weight loss and alleviate non-alcoholic fatty liver disease (NAFLD) via modulation of the gut microbiota. However, key members of the gut microbiota that may mediate the beneficial effects of prebiotics remain elusive. Here, we find that restricted prebiotic feeding during active phase (HF-ARP) induced weight-independent alleviation of liver steatosis and reduced serum cholesterol in high-fat diet (HF) fed mice more significantly than unrestricted feeding (HF-UP). HF-ARP mice also showed concomitantly altered gut microbiota structure that was different from HF-UP group along with significantly increased production of total short-chain fatty-acids (SCFAs). Amplicon sequence variants (ASVs) were clustered into co-abundant groups (CAGs) as potential functional groups that may respond distinctively to prebiotic consumption and prebiotic feeding regime. Prebiotic feeding induces significant alterations in CAG abundances by day 7. Eight of 32 CAGs were promoted by prebiotics, including CAG17 with the most abundant ASV from *Parabacteroides,* CAG22 with *Bacteroides thetaiotamicron* and CAG32 with *Fecalibaculum* and *Akkermansia*. Among the prebiotic-promoted CAGs, CAG20 with ASVs from Lachnospiraceae and CAG21 with ASVs from *Bifidobacterium* and Lachnospiraceae were significantly enhanced in HF-ARP compared to HF-UP. Moreover, most of the prebiotic-promoted CAGs were also significantly associated with improvements in hepatic steatosis, reduction in serum cholesterol and increased cecal propionate production. Together, these results suggest that the impact of prebiotics on weight-independent alleviation of liver steatosis and cholesterol-lowering effect can be optimized by restricting prebiotic intake to active phase and is associated with a distinct change of gut microbiota with increased SCFA production.

## Highlights

• Prebiotics reduced hepatic steatosis and serum cholesterol without weight loss.
• Limiting prebiotic intake to the active phase optimizes the metabolic impact.
• Prebiotic feeding regimes altered the gut microbiota and SCFA production.
• Bacterial guilds promoted by prebiotics positively associated with metabolic health.

## Graphical abstract

**Figure:**
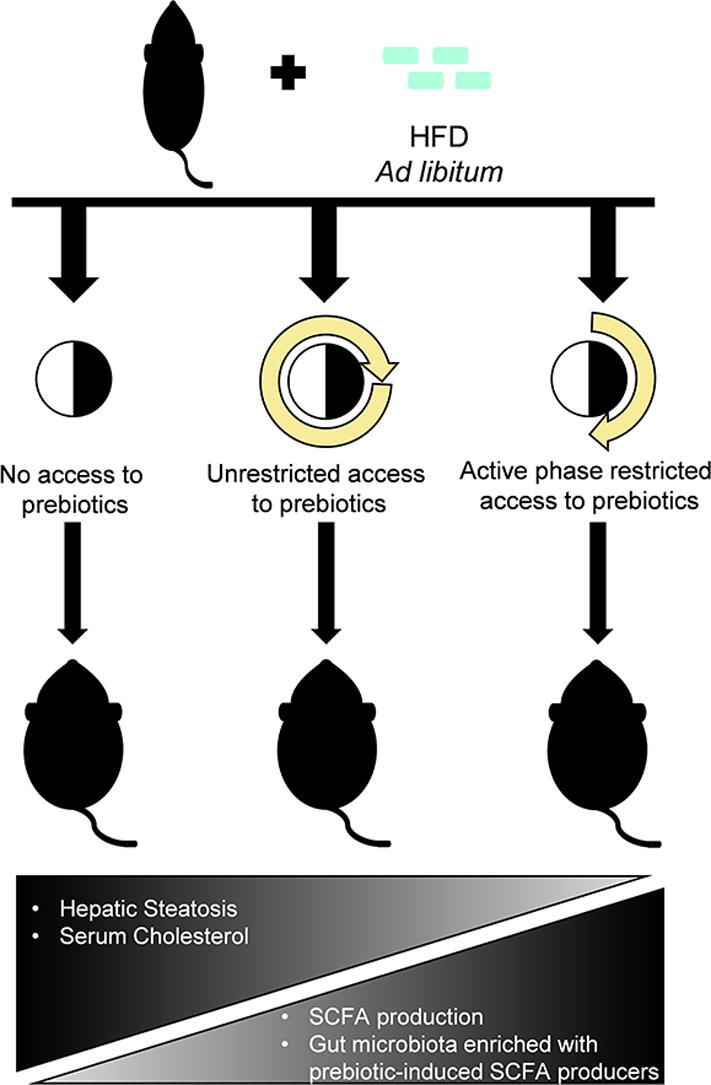

## 1 Introduction

Nonalcoholic fatty liver disease (NAFLD) is currently the most common chronic liver condition and its prevalence has increased in concordance with the obesity epidemic [1], [2]. NAFLD comprises a spectrum of liver conditions starting with steatosis characterized by intrahepatic accumulation of triglyceride and progressing to non-alcoholic steatohepatitis and even hepatocellular carcinoma [1]. To date, weight loss has been considered as the only effective way to manage NAFLD [1], [3]. However, to achieve successful weight loss is challenging and often not sustainable over long-term [4]. Thus, the need for feasible approaches to treat or prevent NAFLD arises.

Growing evidence suggests the importance of gut microbiota in mediating NAFLD [5], [6]. Several studies have established the role of gut microbiota in lipid and cholesterol metabolism by displaying that germfree mice fail to develop NAFLD, even under hypercaloric diet [7], [8], [9], [10]. It was further shown that NAFLD phenotype can be transferred via the gut microbiota by transplanting the gut microbiota from NAFLD-resistant and NAFLD-prone mice into germfree mice, regardless of obesity [11]. Moreover, studies with fecal transplantation involving human donors, further support the involvement of the gut microbiota in NAFLD. In one such study, fecal microbiota transplantation from human donors with hepatic steatosis induced development of hepatic steatosis in mice within a short period [12]. Another study showed that fecal microbiota transplantation from a genetically obese child induced hepatic steatosis in germfree mice fed on normal diet, but when the mice received the fecal microbiota transplant from the same donor after dietary intervention, the mice presented normal liver physiology [13]. Also, NAFLD-associated pathologies developed in high-fat diet (HFD) fed germfree mice inoculated with microbiota from nonalcoholic steatohepatitis patients, instead of healthy donors [14]. Recently, a study demonstrated that nonvirulent endotoxin producing strains of pathobionts overgrowing in the gut of obese human volunteers with severe fatty liver, can induce NAFLD in combination with HFD in germfree mice [15]. These evidence suggest that the gut microbiota plays a fundamental causal role in the development of NAFLD. Consequently, the gut microbiota has become a potential target for improving NAFLD-associated pathologies.

Prebiotics have been shown to alter the gut microbiota, promote the growth of beneficial bacteria, improve obesity-associated metabolic syndrome and NAFLD [6], [16], [17], [18]. Rodent studies have shown that prebiotic feeding can prevent the development of NAFLD-associated pathologies. In one of the earlier studies it was demonstrated that inulin consumption induced weight loss and decreased hepatic triglyceride accumulation in obese rats [19]. In another study, fructo-oligosaccharides supplementation reduced the accumulation of hepatic triglyceride by modulating the gut microbiota, increasing fatty acid oxidation and glucagon-like peptide 1 expression [20]. A variety of prebiotics such as inulin [21], [22], chitin-glucan [23], polysaccharides from fungi [24], arabinoxylan [25] have shown to alleviate NAFLD along with reduction in body weight in HFD-fed mice possibly via production of short chain fatty acids (SCFAs).

Often the reported improvement in NAFLD phenotype is associated with weight loss induced by prebiotic consumption. This makes it difficult to assess the contribution of prebiotic-induced alterations in the gut microbiota in the improvement in NAFLD relative to body weight regulation. Although, the studies mentioned above [19], [20], [22], [23], [24], [25], [21] suggest that prebiotic consumption can induce weight loss and improve NAFLD associated pathologies via modulation of the gut microbiota, the key members of the gut microbiota that may mediate the beneficial effects of prebiotics remain elusive.

In addition, recent studies have shown that the composition and abundance of gut microbiota and its metabolites undergo diurnal oscillations [26], [27]. These microbial oscillations depend on factors such as diet and feeding time [27], [28], [29]. As a result, the gut microbiota would present variable states specific to the time of day for dietary interventions. Prebiotics selectively modulate the gut microbiota and the impact of timed feeding of prebiotics on the gut microbiota and metabolic health has not been studied before. Therefore, in this study we examined if timed feeding of prebiotics can impact the development of NAFLD by interacting with the gut microbiota.

In this study, we evaluate the efficacy of prebiotic feeding regime on gut microbiota and NAFLD-associated pathologies. We find that prebiotic consumption improved the alleviation of hepatic steatosis along with cholesterol-lowering effect, regardless of weight loss. Additionally, we show that restricting prebiotic feeding to active phase of the day can exert a more robust impact on gut microbiota, SCFAs production and metabolic phenotype without the involvement of weight loss.

## 2 Material and methods

### 2.1 Animal experiment and sample collection

**Fig. 1:**
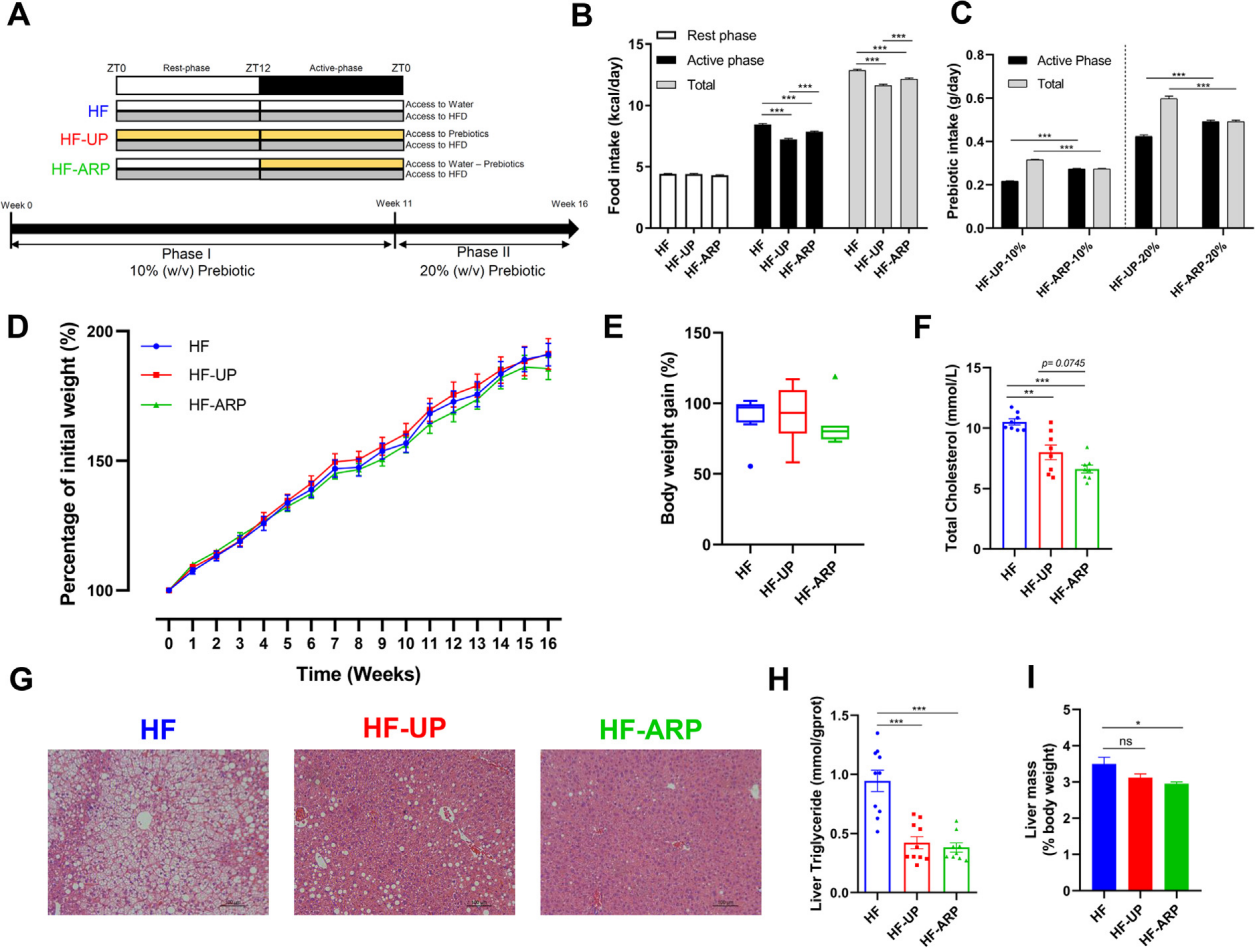
Prebiotic consumption reduces hepatic steatosis and cholesterol levels independent of body weight regulation. (A) Outline of the three different prebiotic feeding regimes used in this study. The three groups have different access to prebiotics; HF = no access to prebiotics, HF-UP = unrestricted access to prebiotics and HF-ARP = active phase (ZT12-ZT0) restricted access to prebiotics. During the experimental period all three groups had *ad libitum* access to HFD. (B) Daily food intake during the study period. (C) Prebiotic intake during the study period. (D) Body weight gain over time. (E) Final body weight gain (n = 10). (F) Quantification of serum cholesterol (n = 8/group). (G) Representative histology images of H&E-stained liver sections. (H) Quantification of hepatic triglyceride (n = 10/group except HF-ARP n = 9). (I) Relative mass of liver (n = 10). Data expressed as mean ± SEM and statistical significance assessed by two-way repeated measures ANOVA (B, D) ANOVA one-way ANOVA (E-F, H-I), followed by a Tukey *post-hoc* test for multiple group comparison and unpaired *t* test for (C) (*p < 0.05, **p < 0.01, ***p < 0.001).

Specific pathogen-free (SPF), 5-week-old male mice (C57BL6/J) were procured from SLAC Inc. (Shanghai, China) and acclimated at the animal center under a strict 12 h light: 12 h dark cycle, with lights being turned on from 7 a.m. to 7 p.m. (Zeitgeber time: ZT 0 denotes lights on and ZT12 denotes light off) with normal control diet (NCD, AIN93G Research Diet, NJ, USA) available *ad libitum* for 3 weeks with 5 mice per cage at constant temperature (22 °C ± 3 °C) with access to autoclaved water. After the acclimation phase the mice were randomly assigned to one of the following three groups as described in Fig. 1A: (i) control group with no access to prebiotic (HF, n = 10), (ii) unrestricted access to prebiotic (HF-UP, n = 10) and (iii) active phase (ZT12-ZT0) restricted access to prebiotic (HF-ARP, n = 10). During the experimental period all three groups had *ad libitum* access to HFD (D12492, 60% kcal fat Research Diet, NJ, USA, Table S1). The prebiotic Formula 3 used in this study was manufactured by Perfect (China) Co., Ltd and is a mixture of resistant starch, fructooligosaccharide, inulin and xylooligosaccharide (Table S2). The prebiotic was administered via drinking water at 10% (w/v) for 11 weeks followed by 20% (w/v) for 4 weeks. The prebiotic solution was prepared fresh at the beginning of each phase of the day and distributed according to the feeding regime described in Fig. 1A and at the same time the cage of the mice was changed. During the study period, food and water intake was measured twice daily. Body weight was measured twice per week and at the same time every week. At the end of the study period, mice were sacrificed after 6 h of fasting followed by collection of serum and tissue samples, which were stored at −80 °C until further analysis. Fresh fecal samples (2–3 fecal pellets/mice) were collected from the mice individually and stored at −80 °C until DNA extraction. All animal experiments were approved by the Institutional Animal Care and Use Committee (IACUC) of the School of Life Sciences and Biotechnology of Shanghai Jiao Tong University (No. 2018035).

### 2.2 Serum cholesterol measurement

Levels of serum total cholesterol was measured using enzymatic colorimetric assay kit according to the manufacturer’s instructions (Nanjing Jiancheng Bioengineering Institute, China).

### 2.3 Liver histology and hepatic lipid measurement

Specific sections of the fresh liver from each mouse were fixed in paraformaldehyde. The samples were then embedded in paraffin and subsequently sectioned and stained with hematoxylin and eosin. The samples were observed and images were captured using Leica DMRBE microscope. Hepatic triglyceride was measured as described previously [13] using an assay kit according to the manufacturer’s instructions (Nanjing Jiancheng Bioengineering Institute, China).

### 2.4 Measurement of SCFAs

The concentrations of SCFAs was measured in the cecal content collected from the mice, by mixing 0.2 g of the cecal content in 1 ml PBS to prepare a homogenous mixture and then centrifuged at 16000*g* for 15 min at 4 °C. Next, the supernatant was filtered through 0.22-µm filter. Then, 200 μl of the filtrate was acidified using 0.1 ml of 50% (v/v) sulfuric acid and extracted using 0.4 ml of diethyl ether. Consequently, concentration of SCFA (acetic acid, propionic acid and butyric acid) was determined on Agilent 6890 gas chromatography system coupled with flame ionization detector using a polar HP-FFAP (0.25 mm × 0.25 mm × 30 m) capillary column (Agilent Technologies, USA). Hydrogen gas was used as carrier gas, with a flow rate of 1.0 ml/min. The oven temperature was initially set at 140 °C for 10 min, then increased to 165 °C at 5 °C/min and then to 270 °C at 25 °C/min for 2 min. The detector temperature was set at 280 °C and the inlet temperature at 250 °C. Sample volume of 5 μl was used and running time for each sample was 30 min. SCFAs were quantified by comparing peak areas with those of chemical standards and data was acquired with the Agilent ChemStation (version G2070AA, Agilent Technologies).

### 2.5 Microbial DNA extraction and 16S rRNA gene V3-V4 region sequencing

Microbial DNA was extracted from the fecal samples collected from the individual mice on the day before the start of the prebiotic feeding regime for baseline/day 0 timepoint and subsequently 7, 77, 84 and 105 days after initiating the prebiotic feeding regime to explore the variation of gut microbiota during the study period, according to a method described previously [30]. A total of 149 samples were sequenced (HF, *n* = 10; HF-UP, *n* = 10; HF-ARP, *n* = 10 for each timepoint except HF-UP d7, n = 9). The V3-V4 regions of 16S rRNA gene amplicons were sequenced on the MiSeq instrument (Illumina Inc., USA). A 16S rRNA gene sequencing library for the V3-V4 regions was prepared according to a modified version of the instructions provided by the manufacturer [31]. Briefly, for the amplicon PCR; 25-μl reaction mix for each DNA sample was prepared containing 2x Phanta Max Buffer, 10 mM dNTP Mix, 1 μM of each specific primer for V3–V4 region of 16S rRNA gene as described in protocol, 0.5 U of Phanta Max Super-Fidelity DNA polymerase (Vazyme, China) and 10 ng microbial DNA. PCR was performed according to the following program: a pre-denaturation step at 95 °C for 3 min followed by 21 cycles of denaturation (30 s at 95 °C), annealing (30 s at 55 °C), extension (30 s at 72 °C) and a final extension step (5 min at 72 °C). Then, PCR products were detected by 1.2% agarose gel electrophoresis and purified by Hieff NGS® DNA Selection Beads (Yeasen Biotech Co. Ltd., China) beads followed by detection of purified PCR products by 1.2% agarose gel electrophoresis. Next, for the index PCR (Nextera XT Index Kit); 25-μl reaction mix contained 2x Phanta Max Buffer, 10 mM dNTP Mix, 2.5 μl of each Index primers (Nextera XT Index F-indexing primer and R-indexing primer), 0.5 U of Phanta Max Super-Fidelity DNA polymerase, and 2.5 μl of purified PCR product from the previous step. PCR was performed according to the following program: a pre-denaturation at 95 °C for 3 min followed by 8 cycles denaturation (30 s at 95 °C), annealing (30 s at 55 °C), extension (30 s at 72 °C) and a final extension step (5 min at 72 °C). The purification of index PCR products was carried out with Hieff NGS® DNA Selection Beads according to the protocol and detected by gel electrophoresis. The purified products were mixed at an equal ratio, quantitated by Qubit (Life Technologies, United States) and gel electrophoresis to ensure correct amplicon size. The final pooled sample library was denatured with NaOH, diluted to 12 pM with hybridization buffer Illumina-HT1, spiked with 30% PhiX and then heat denatured at 96 °C for 2 min prior to loading it onto a MiSeq v3 reagent cartridge and sequenced using the Illumina Miseq System (Illumina Inc., United States).

### 2.6 Microbiota data analysis

The 16S rRNA gene sequence data was processed and analyzed on the QIIME2 software (v2018.11) [32]. The raw sequence data was demultiplexed and then denoised with DADA2 pipeline (q2‐dada2 plugin) [33] to obtain the amplicon sequence variants (ASVs) frequency data table. Alpha diversity metrics (Observed ASVs and Shannon’s index), beta diversity metric (Bray-Curtis dissimilarity), and Principle Coordinate Analysis (PCoA) were performed using the q2-diversity after rarefying the samples to 16,500 sequences per sample. One sample was excluded from downstream diversity analysis as the number of high-quality reads obtained was less than 16,500. The alpha diversity indices were compared at each timepoint with Kruskal-Wallis tests and the *p* values were adjusted for multiple comparison with Benjamini-Hochberg method [34]. The structure of the gut microbiota was compared using beta diversity with Bray-Curtis dissimilarity metric, visualized by principal-coordinate analysis (PCoA) plots and statistical significance assessed by permutational multivariate analysis of variance (PERMANOVA) via q2-diversity with 9,999 permutations and *p* values were adjusted with Benjamini-Hochberg correction method [34]. Taxonomic assignment for ASVs was performed via the q2-feature-classifier [35] using the SILVA rRNA gene database [36].

### 2.7 Microbial CAG network analysis

Co-abundant group (CAG) network analysis was used to identify the key responsive phylotypes, which were associated with improvements in hepatic steatosis and cholesterol-lowering effect. The correlations between the ASVs which were shared among at least 20% of the samples were calculated by using the SparCC method (bootstrap value, 100) [37]. Next, on the basis of SparCC correlation coefficient matrix, SparCC distance matrix (1-SparCC correlation coefficient) was calculated for the 275 shared ASVs, which were then clustered into 32 CAGs by applying Ward’s hierarchical clustering method and PERMANOVA with 999 permutations. A cluster tree was constructed and PERMANOVA was applied sequentially along the tree from top to bottom to identify the nodes with no significant difference as a single CAG (*p >* 0.001). Then, the CAG network was visualized in Cytoscape v3.8.0 [38].

### 2.8 Statistical analysis

Statistical analysis was performed using Graphpad Prism 8 software and R (version 3.6.2). The following R packages were used for analysis; ComplexHeatmap [39] and ggplot2. One-way analysis of variance (ANOVA) followed by Tukey post hoc test for multiple group comparison was used to compare the HF, HF-UP, and HF-ARP for physiological and biochemical data. Body weight gain over time was compared using two-way repeated measures ANOVA. Prebiotic intake was compared using unpaired *t*-test. Spearman correlation analysis was used to determine the association between CAGs, metabolic parameters and SCFAs followed by FDR correction using the Benjamini and Hochberg. Differential abundance of CAGs was tested by the Mann-Whitney test followed by FDR correction using the Benjamini-Hochberg method.

### 2.9 Data availability

The 16S rRNA gene sequence data generated in this study has been submitted to Sequence Read Archive (SRA) maintained by NCBI under the accession number PRJNA657015.

## 3 Results

### 3.1 Prebiotic consumption improved hepatic steatosis and lowered serum cholesterol independent of weight loss

During the study period, despite similar weight gain in all three groups (Fig. 1D and E), elevated levels of cholesterol (Fig. 1F), and hepatic steatosis (Fig. 1G and H) was only observed in HF group. In contrast, both HF-UP and HF-ARP groups displayed improvement in hepatic steatosis (Fig. 1G), and this improvement was confirmed by measuring hepatic triglyceride (Fig. 1H). Interestingly, even though both HF-UP and HF-ARP showed reduction in liver weight compared to HF but only HF-ARP showed significant reduction (Fig. 1I). Hepatic triglyceride was reduced by 55.4% in HF-UP and 59.7% in HF-ARP compared to HF group. Additionally, both HF-UP and HF-ARP exhibited cholesterol-lowering effect, but HF-ARP showed larger improvement, where total cholesterol was reduced by 24% in HF-UP and 37.1% in HF-ARP compared to HF group (Fig. 1F). Also, in comparison with HF-UP, HF-ARP showed further reduction in cholesterol levels (*p = 0.0745*). Interestingly, prebiotic-induced improvement in hepatic steatosis and cholesterol-lowering effect observed in this study were independent of weight loss.

Overall, consumption of prebiotics significantly reduced the daily food intake, particularly during the active phase (Fig. 1B). Also, HF-ARP consumed more food than HF-UP (Fig. 1B). However, this reduction in daily food intake did not alter the cumulative food intake significantly (Fig. S1). Total daily prebiotic intake was significantly higher for HF-UP, however active phase specific consumption of prebiotic was higher in HF-ARP (Fig. 1C). During the phase I of the study period with 10% (w/v) prebiotic consumption we did not see an impact on the body weight (Fig. 1D) and subsequently increased the prebiotic consumption to 20% (w/v) in phase II. However, with increased prebiotic consumption (Fig. 1C), we did not see an impact on the body weight (Fig. 1D). We did not observe biologically relevant cage-dependent confounding effect of food or prebiotic intake on body weight gain (Fig. S8, Tables S3 and S4).

### 3.2 Restricting the feeding time of prebiotics to the active phase increased SCFA production

**Fig. 2:**
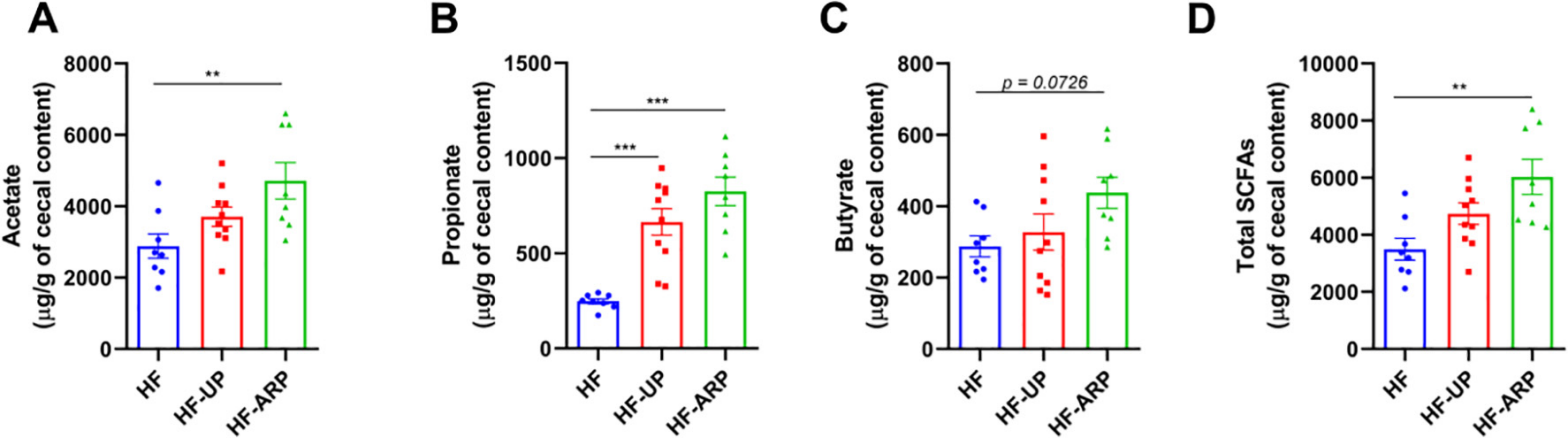
Active phase restricted feeding of prebiotics results in increased production of SCFAs in HFD-fed mice. Quantification of cecal acetate (A), propionate (B) butyrate (C) and total SCFA (D) production (n = 8/group except HF-UP n = 10). Data expressed as mean ± SEM and statistical significance assessed by one-way ANOVA, followed by a Tukey *post-hoc* test for multiple group comparison (*p < 0.05, **p < 0.01, ***p < 0.001).

Here, we found that HF-ARP mice displayed a significant increase in overall SCFA production (Fig. 2D). Specifically, propionate production was increased in both HF-UP and HF-ARP (Fig. 2B). However, acetate (p < 0.01) and butyrate (p = 0.07) were increased in HF-ARP (Fig. 2A and C). Moreover, increase in SCFA production, particularly propionate, was associated with improvement in hepatic steatosis (Fig. S6). Also, we found that the prebiotic treatment significantly increased the colon length and weight (Fig. S2A and B). Furthermore, the prebiotic treatment significantly increased cecum weight and cecal tissue weight (Fig. S2C and D). These indicate that prebiotic feeding prevented the loss of colonic and cecal mass.

### 3.3 Gut microbiota structure was altered by both prebiotic consumption and prebiotic feeding regimen

**Fig. 3:**
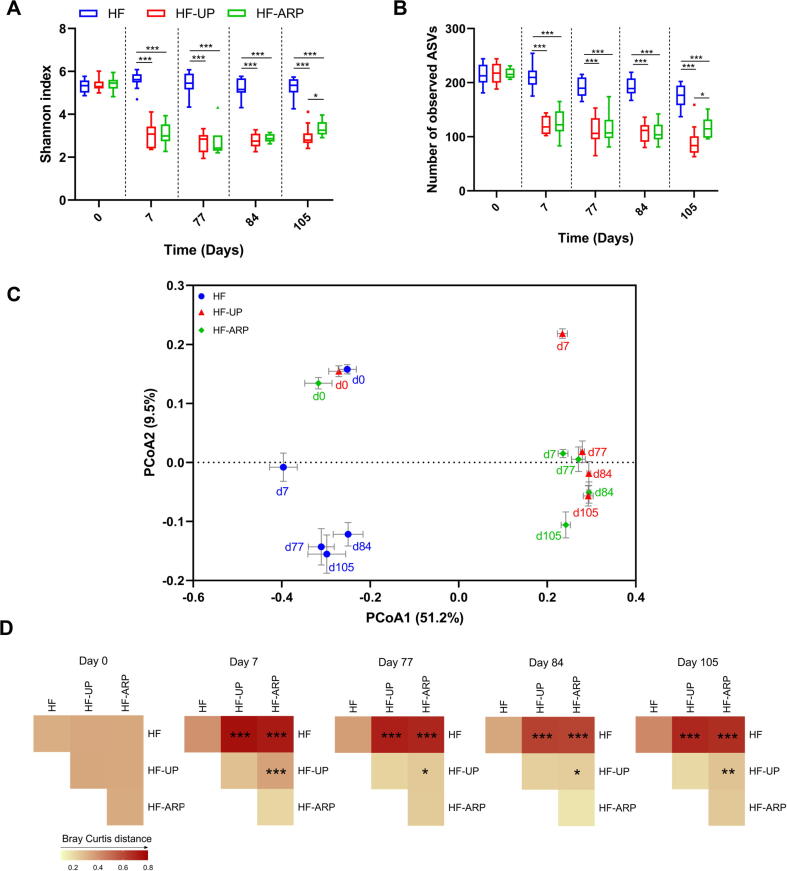
Active phase restricted feeding of prebiotics modulates the structure of gut microbiota differently than unrestricted access in HFD-fed mice. (A) Alpha diversity measured by Shannon index and (B) Observed ASVs index. Data expressed as mean ± SEM and compared at each timepoint with Kruskal-Wallis tests and *p* values adjusted with Benjamini-Hochberg correction for multiple group comparison, *p < 0.05, ***p < 0.001. (C) Principal-coordinate analysis (PCoA) plot based on Bray-Curtis dissimilarity index. Each point represents the mean score along with SEM for each group and timepoint (represented by d0, d7, d77, d84 and d105) (D) Heatmap of Bray-Curtis index between each group along with corresponding PERMANOVA comparisons (9,999 permutations) at each timepoint (left to right), and *p* values adjusted with Benjamini-Hochberg correction for multiple group comparison, *p < 0.05, **p < 0.01, ***p < 0.001.

To analyze the structural changes of the gut microbiota in response to both prebiotic consumption and prebiotic feeding regimen, we sequenced the V3-V4 region of the 16S rRNA gene using the fecal samples collected from the mice on the day before the start of the prebiotic feeding regime for baseline/day 0 timepoint and subsequently days 7, 77, 84 and 105 after initiating the prebiotic feeding regime to explore the variation of gut microbiota during the study period. After sequencing, we obtained 4,760,878 high-quality reads and 853 ASVs, with an average of 31,952 ± 5799 reads per sample. The alpha diversity (Fig. 3A and B) of the gut microbial community was significantly reduced in HF-UP and HF-ARP compared to HF group after only 7 days of prebiotic feeding and continued to be significantly reduced throughout the study duration. There was no difference in gut microbial alpha diversity indices between HF-UP and HF-ARP until day 84, when HF-ARP showed an increase in diversity compared to HF-UP.

Before the start of prebiotic consumption, the three groups displayed no significant difference in the gut microbiota structure (Fig. 3C and D). The overall structure of gut microbiota was significantly altered in HF-UP and HF-ARP groups compared to HF group only after 7 days of prebiotic consumption and continued to display significant differences throughout the study duration, as shown by the PCoA (Fig. 3C and S7) and PERMANOVA analysis (Fig. 3D) based on the Bray-Curtis dissimilarity matrix. This indicates that consumption of prebiotics has a strong impact on the structure of gut microbiota starting within a short duration. Moreover, the difference in prebiotic feeding regime also displayed a significant impact on the gut microbiota structure starting as early as day 7. However, the magnitude of dissimilarity index between HF-UP and HF-ARP decreased over time but was still significantly different as shown in Fig. 3C and E.

Together, these results suggest that both prebiotics and difference in prebiotic feeding regime exert a significant effect on the overall gut microbiota structure in a relatively short duration, long before alleviation of metabolic phenotypes were observed.

### 3.4 CAGs were associated with improvements in hepatic steatosis, cholesterol levels and production of cecal SCFAs

**Fig. 4:**
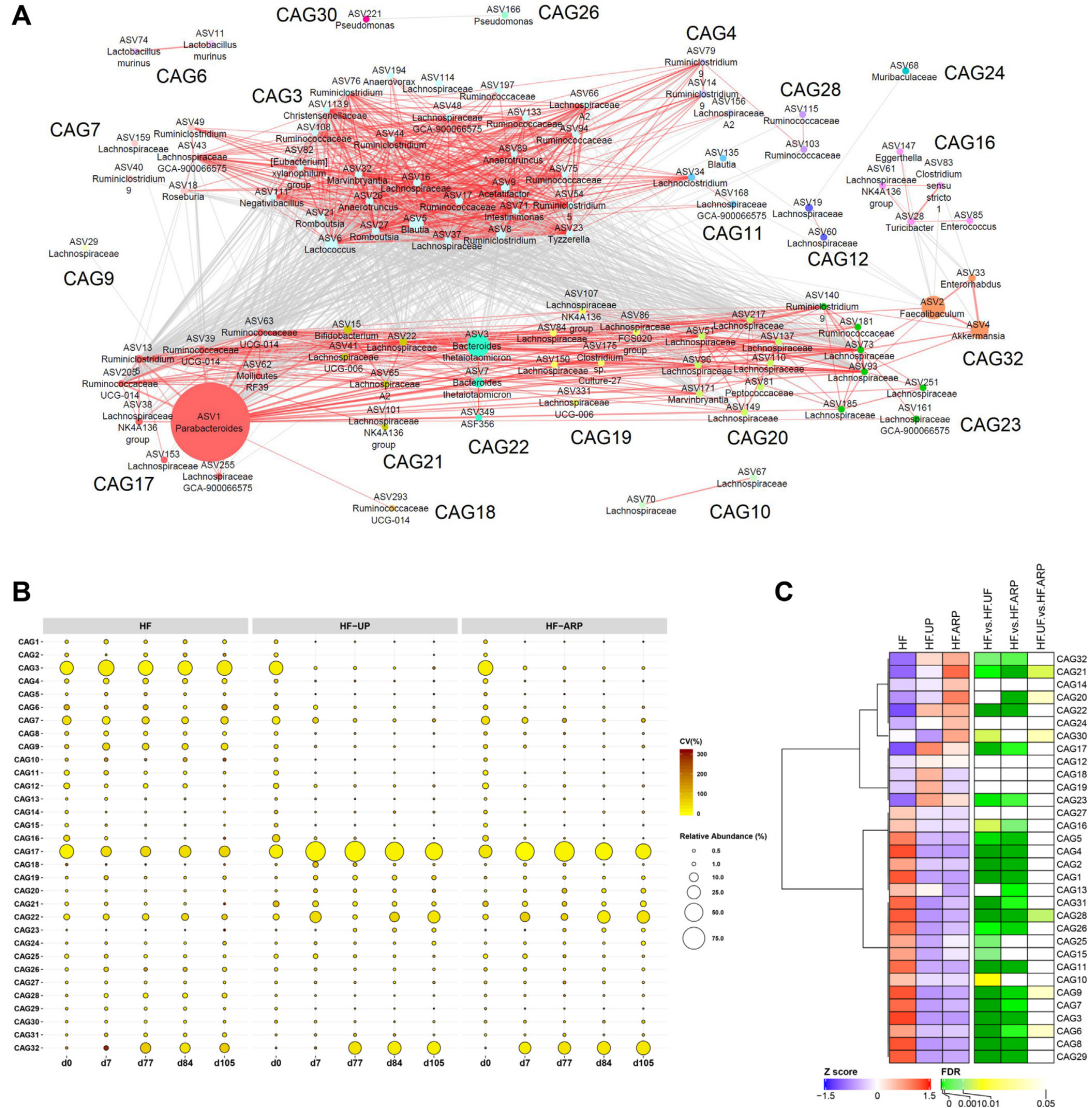
Alterations in the abundance of CAGs in response to both consumption of prebiotics and prebiotic feeding regime*.* (A) Microbial interaction network displays the interaction between different CAGs. Node size represents the mean abundance of ASV. The edges between the nodes indicate correlation (grey = negative, red = positive), with width of the edge corresponding to magnitude of the correlation. Correlations with absolute values less than 0.6 are not shown here. (B) Bubble plot shows the variation in the abundance of CAGs over time. The size and color of the circles represent mean relative abundance and coefficient of variance of each CAG respectively. (C) Heatmap for the mean relative abundance for each CAG for day 105, are expressed as *Z* scores along with FDR values for comparison between the groups using Mann-Whitney test followed by FDR correction using Benjamini and Hochberg procedure. CAGs were ordered by Spearman’s correlation analysis based on their relative abundance. (For interpretation of the references to color in this figure legend, the reader is referred to the web version of this article.)

To identify the key members of the gut microbiota which respond to the prebiotic feeding regime as a functional group, we used co-abundant analysis to identify potential functional groups as guilds. A microbial co-abundant network was built to visualize the plausible interactions between the 275 ASVs which were shared by at least 20% of the samples, and accounted for 97.18 ± 2.14% (mean ± S.D.) of the total sequences in each sample. The ASVs were further clustered into 32 co-abundant groups (CAGs) by SparCC method and PERMANOVA analysis (Fig. 4A).

Consumption of prebiotics altered guild-level organization of the gut microbiota as early as day 7 of prebiotic feeding (Fig. 4B, S3A and S3B). At day 7, four CAGs including, CAG17, CAG20, CAG21, and CAG22 were significantly increased in response to prebiotic consumption in both HF-UP and HF-ARP, while CAG32 was significantly increased only in HF-ARP. Also, CAG18 and CAG19 were significantly increased only in HF-UP at day 7 and remained significantly enhanced compared to HF-ARP over the course of study duration except for day 105. At day 77, CAG32 was increased in all three groups, whereas at day 84 and 105 it was significantly increased in HF-ARP and HF-UP compared to HF. CAG20 started showing significant difference between HF-UP and HF-ARP from day 84 and continued to show significant difference also at day 105. Furthermore, CAG21 was significantly increased in HF-ARP compared to HF-UP at day 105, while CAG17 was enhanced in HF-UP compared to HF-ARP at day 105. Additionally, CAG23 increased in response to prebiotic feeding at day 77 and showed significant increase over time. Overall, eight CAGs were promoted by prebiotic feeding and six of them (CAG17, CAG18, CAG19, CAG20, CAG21 and CAG32) showed differential enrichment between HF-UP and HF-ARP over the course of the study duration. On the other hand, consumption of prebiotic significantly decreased the relative abundance of fifteen CAGs after 7 days of prebiotic feeding, these CAGs remained significantly decreased throughout the study period (Fig. 4B).

**Fig. 5:**
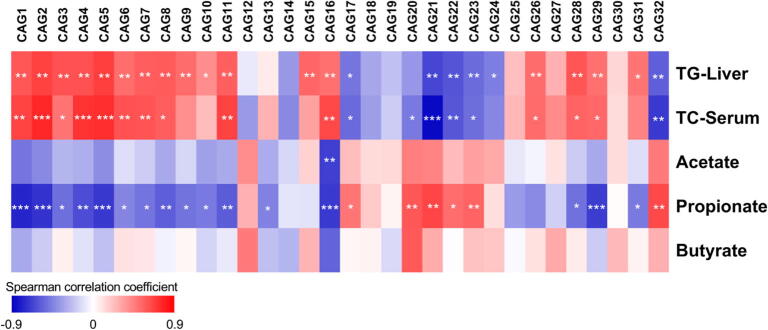
Associations between CAGs, metabolic phenotype and cecal SCFAs. Heatmap of Spearman’s correlation coefficients (with FDR correction) between relative abundance of CAGs for day 105 and hepatic triglyceride (TG-Liver), serum total cholesterol (TC-Serum) and cecal SCFAs. *FDR < 0.05, **FDR < 0.01, ***FDR < 0.001.

As hepatic triglyceride and serum cholesterol were measured at the end of the study period, we compared the relative abundance of CAGs between the three groups at day 105 (Fig. 4C). At day 105, five CAGs including, CAG17, CAG21, CAG22, CAG23 and CAG32 were significantly increased in response to prebiotic consumption in both HF-UP and HF-ARP, while CAG20 was only increased in HF-ARP. These five CAGs were promoted by prebiotic feeding and also showed significant negative correlation with levels of hepatic triglyceride and serum cholesterol (Fig. 5). Interestingly, these five CAGs were also strongly associated with propionate production in the cecum (Fig. 5).

Among these CAGs, CAG17 was the dominant CAG with ASVs from *Parabacteroides,* Lachnospiraceae, Ruminococcaceae and *Mollicutes,* displaying interaction between ASVs from different phyla within the same functional guild. Also, CAG 17 was enriched in HF-UP when compared to HF-ARP (FDR = 0.0518) (Fig. 4C, S3A and S3B). CAG21, consisting of ASVs from *Bifidobacterium* and Lachnospiraceae was significantly enriched in HF-ARP compared to HF-UP (Fig. 4C, S3A and S3B), demonstrating that the prebiotic feeding regime can regulate the impact of prebiotic on the gut microbiota. CAG22, comprised predominantly of *Bacteroides thetaiotamicron*, was not significantly different between HF-UP and HF-ARP (Fig. 4C, S3A and S3B). Similarly, CAG23 composed of ASVs from Lachnospiraceae, Ruminococcaceae and *Ruminiclostridium* and CAG32 with ASVs from *Fecalibaculum*, *Akkermansia*, and *Enterorhabdus* genus were not significantly different between HF-UP and HF-ARP (Fig. 4C, S3A and S3B). Moreover, these five CAGs along with CAG20 were positively correlated with each other (Fig. 4A).

Additionally, CAG20 with 8 ASVs from Lachnospiraceae and one each from *Peptococcaceae* and *Marvinbryantia* (Fig. S3B), was enriched in HF-ARP and significantly correlated with improvement in cholesterol levels and modest improvement in hepatic triglyceride levels (FDR = 0.0666) (Fig. 5).

Fifteen CAGs including CAG1, CAG2, CAG3, CAG4, CAG5, CAG6, CAG7, CAG8, CAG9, CAG11, CAG16, CAG26, CAG28, CAG29, CAG31 were significantly decreased in response to prebiotic consumption in both HF-UP and HF-ARP, while CAG10, CAG15, CAG25 and CAG30 were only decreased in HF-UP and CAG13 in HF-ARP (Fig. 4B and C). Among the CAGs, which decreased in response to prebiotic consumption, seventeen CAGs were positively associated with hepatic triglyceride and thirteen CAGs with serum cholesterol (Fig. 5). Most of these CAGs, also correlated negatively with propionate production in the cecum (Fig. 5). CAG3 was the dominant CAG with 29 ASVs, with members from *Blautia, Lactococcus, Acetatifactor,* Ruminococcaceae, *Ruminiclostridium,* Lachnospiraceae, *Anaerotruncus, Tyzzerella, Romboutsia, Marvinbryantia,* which decreased in response to prebiotic consumption.

Interestingly, in addition to CAG 20 and CAG21 described earlier as significantly enriched in HF-ARP compared to HF-UP (Fig. 4C and S4), four more CAGs which decreased in response to prebiotic consumption were significantly increased in HF-ARP compared to HF-UP (Fig. 4C and Fig. S4). CAG6, which contained two ASVs from *Lactobacillus murinus*, along with one each from *Frisingicoccus caecimuris* and *Streptococcus salvarius*. Among these ASVs, relative abundance of ASV11 *Lactobacillus murinus* and ASV57 *Frisingicoccus caecimuris* were increased in HF-ARP compared to HF-UP (Fig. S4). Within CAG9 relative abundance of ASVs from *Intestinimonas* and Lachnospiraceae were significantly increased in HF-ARP compared to HF-UP (Fig. S4). Also, in CAG28 relative abundance of ASV from *Intestinimonas* and within CAG30, relative abundance of ASVs from Lachnospiraceae and Ruminococcaceae were increased in HF-ARP compared to HF-UP (Fig. S4).

Together, these results show that prebiotic consumption alone can significantly impact the gut microbiota composition within a short duration, nevertheless the difference in prebiotic feeding regime also has a modulating effect.

## 4 Discussion

In this study, we report that (i) prebiotic consumption can alleviate hepatic steatosis and lower cholesterol level regardless of weight loss; (ii) active phase restricted feeding of prebiotics showed more significant effects on modulating gut microbiota, SCFA production and metabolic response, independent of weight loss; (iii) CAGs promoted by prebiotic feeding were significantly associated with improvements in hepatic steatosis and cholesterol-lowering effect.

Previous studies have shown prebiotic-induced alleviation of NAFLD is dependent on achieving successful weight loss [19], [20], [21], [22], [23], [24], [25]. In contrast, in the current study we show that prebiotic consumption can improve hepatic steatosis in HFD-fed mice irrespective of weight loss which suggests that the prebiotics induced alterations in the gut microbiota might directly impact liver lipid metabolism via the gut-liver axis. This difference in response might partly be attributed to the differential capability of the initial gut microbiota composition to utilize the prebiotic, which in turn gives rise to an altered metabolite profile such as SCFAs. Moreover, recent studies have reported variable metabolic response including adverse reaction to prebiotic consumption, which is dependent on the composition of the baseline/preintervention gut microbiota with variable prebiotic metabolizing capacity [40], [41], [42], [43]. Additionally, a recent study reported that discrete structural differences in resistant starch can alter the gut microbiota selectively to either produce butyrate or propionate [44]. Nevertheless, our current study allows us to explore prebiotic-induced changes in gut microbiota which might be specific for improvements in hepatic steatosis independent from weight loss. Also, the mechanisms involved in metabolic response in weight loss-dependent and weight loss-independent improvements in NAFLD might be different and need further evaluation.

So far, the mechanisms by which gut microbiota impacts the liver health and metabolism is poorly understood. However, the gut-liver axis via the portal circulation enables the transport of nutrients and metabolites produced by the gut microbiota. Our data suggests that the improvement in HFD induced hepatic steatosis by prebiotics could be associated with the increased production of SCFAs. Here, in addition to these two factors, our findings suggest that SCFA production can also be modified by timed feeding of prebiotics. This implies that the distinct alterations in the gut microbiota introduced by the difference in prebiotic feeding regime might be an outcome of the gut microbiota undergoing diurnal oscillation [27]. The molecular mechanisms of prebiotic-induced improvement of hepatic steatosis is still incompletely understood. However, prior studies suggest that prebiotics and SCFAs, particularly propionate, may reduce accumulation of triglyceride by regulating the expression of genes involved in lipogenesis, fatty acid uptake and oxidation [11], [19], [40], [45], [46], [47]. One such pathway involves the inhibition of the genes in de novo lipogenesis pathways such as acetyl-coenzyme A carboxylase (ACC) and fatty acid synthase (FAS) [45], [47]. Another pathway involves SCFA-induced repression of peroxisome proliferator–activated receptor-γ (PPARγ) activity, which via a cascade of reactions stimulates fatty acid oxidation [46]. Still, the current understanding of the key members of the gut microbiota that may mediate the beneficial effects of prebiotics remain elusive. Here, we observed that differences in prebiotic feeding regime altered the structure and composition of fecal microbiota differently over time and the CAGs promoted by prebiotic were positively correlated with both improvement in hepatic steatosis and increased propionate production. Interestingly, prebiotics selectively enriches a small limited subset of ASVs compared to the large number of them available in the gut, suggesting that only a few gut bacteria have the ability to participate in the metabolic degradation and utilization of prebiotics. Prebiotic-promoted CAG17 is composed of the most abundant ASV from *Parabacteroides*, ASVs from *Ruminiclostridium 5*, Lachnospiraceae and Ruminococcaceae. Recently, two different species from the genus *Parabacteroides* have been reported to alleviate obesity, hyperglycemia and hepatic steatosis in HFD-fed mice [48]; and members of this genus are enriched in the gut in response to prebiotic in mice [24], [49] and humans [44]. Additionally, members of *Parabacteroides* genus encode the metabolic pathways for succinate production, which can be converted to propionate [48]. Lachnospiraceae and Ruminococcaceae members have been reported to produce butyrate and propionate in the gut [50]. Additionally, the members of the remaining prebiotic-promoted CAGs such as *Bacteroides thetaiotamicron*, *Akkermansia*, *Bifidobacterium* can potentially produce SCFAs [51], [52]. As mentioned earlier, we identified distinct alterations in the gut microbiota which were specific to the two prebiotic feeding regimes. Particularly, increase in CAGs containing Lachnospiraceae, Ruminococcaceae, *Bifidobacterium*, and *Intestinimonas* in the active phase restricted prebiotic fed group may in part contribute to the increased production of SCFAs.

On the other hand, prebiotic consumption also decreases the relative abundance of several CAGs, and some of these CAGs were also associated positively with hepatic steatosis and higher cholesterol levels. For example, CAG3 that contains members of *Blautia*, and other Lachnospiraceae, known SCFA producers [50], are negatively associated with improvements in metabolic phenotype in this study. However, few studies have also found increased abundance of members of the Lachnospiraceae family in mice that develop hepatic steatosis [11] and in NAFLD patients [53]. Here, we observed that ASVs assigned to the same taxonomic group could behave differently in response to the same treatment, such as Lachnospiraceae. Genomic intra-species diversity can account for up to 30% difference in bacterial strains belonging to the same species, which can result in differences in their functions [54]. Based on comparative genomics, core-genome encoded functions are shared by all members of a species whereas strain-specific functions are encoded in the pan-genome where either a single strain or some strains contain the genes [55]. Thus, members belonging to the same taxon often do not function similarly [56], and analysis carried out at the highest resolution available (ASV) will be adequate in such cases. Bacteria in the gut do not occur in isolation, they interact with each other at strain-level forming coherent functional groups termed “guilds” [57]. Guilds are assembled by clustering together individual members based on co-abundance patterns [58]. This allows members within an ecosystem to interact with each other and form a guild solely based on their ability to use a resource or otherwise, such as prebiotics in our study, regardless of their taxonomic affiliation. Also, guild-based approach can help in reducing the problem of dimensionality associated with microbiome data by identifying functionally relevant members, which can be explored further. In comparison to a taxon-based analysis, where different members of a taxon function in an unrelated manner, guild-based approach offers an ecologically relevant tool to identify the key members of gut microbial community associated with a particular host phenotype. Accordingly, to understand the response of gut microbiota to prebiotics, an ecological perspective is essential, as prebiotic fermentation is driven by complex interactions between members of the gut microbiota via cross-feeding across taxonomic backgrounds [59], [60]. Here, in the current study CAG analysis allows for a more suitable method to understand the prebiotic-induced alterations in the gut microbiota. Thus, prebiotic feeding may benefit the host via promotion of functional groups of gut bacteria that can produce SCFAs, and this beneficial effect on host metabolism can be further optimized by restricting prebiotic intake to the active phase in HFD-fed mice. Further studies would be required to understand causal links between the selectively promoted gut microbes and improved hepatic steatosis.

There have been few studies, which show that prebiotics can influence serum cholesterol levels in mice [20], [61] and humans [62]. Additionally, few cholesterol-reducing bacterial strains from diverse taxonomic groups, such as *Bacteroides* genus, *Eubacterium coprostanoligenes and Lactobacillus* genus, have been isolated [63], [64], [65]. Recently, a group of microbial cholesterol dehydrogenases, that participates in the transformation of cholesterol into coprostanol, which is eliminated in feces, was identified in the gut microbiome and was reported to be associated with cholesterol-lowering effects [66]. Moreover, fecal transplant from humans with elevated plasma cholesterol levels into microbiota-depleted mice induced hypercholesterolemia [67]. Together, these studies show that the gut microbiome is involved in cholesterol metabolism. However, the members of the gut microbiota that mediate or influence the cholesterol metabolism is poorly understood. Among the SCFAs, propionate has been found to be associated with cholesterol-lowering effects, but the mechanisms still needs to be elucidated [61], [62]. The results from our study showed that prebiotic-induced alterations in gut microbiota were strongly associated with both cholesterol-lowering effects and increased propionate production. Based on these findings, we suggest that the members of prebiotic-promoted CAGs are associated with cholesterol-lowering effect via SCFA production (propionate and acetate). Additional studies would be required to understand the causal links between these prebiotic-induced gut microbes and cholesterol-lowering effect.

Lack of treatment modalities for NAFLD have made prebiotic-induced selective modulation of gut microbiota an interesting target in improving NAFLD-associated pathologies. As NAFLD is a progressive liver disease, by preventing the development of hepatic steatosis, prebiotics could potentially prevent further progression or susceptibility to liver inflammation and injury. Also, cholesterol is lipotoxic and can induce NASH [68]. Recently, a cholesterol-lowering drug was reported to prevent the development of NAFLD-associated liver cancer by modulating the gut microbiota, thus the cholesterol-lowering effect of prebiotic reported in this study might potentially help in preventing liver injury [68]. Taken together, prebiotics may benefit the host by improving hepatic steatosis and lowering cholesterol independent of weight loss via selective promotion of functional groups of gut bacteria that can produce SCFAs. This can also potentially help in lowering the susceptibility to liver injury, which needs further studies.

There are limitations to the current study, first, the small difference in prebiotic intake between the two prebiotic feeding groups. Interestingly, despite a lower prebiotic intake, active phase restricted feeding of prebiotics produced increased amounts of total SCFAs, suggesting that SCFA production efficiency can be improved by timed feeding of prebiotics. Second, not measuring lipids excreted in fecal samples over 24 h duration. These can be helpful in designing further studies.

In summary, prebiotic consumption induced weight loss independent alleviation of hepatic steatosis and cholesterol-lowering effect. Prebiotics had a profound impact on the gut microbiota structure and composition within a short duration. Furthermore, we reported that restricting the feeding of prebiotics to the active phase can further optimize the impact of prebiotics on gut microbiota, SCFA production, and overall metabolic response independent of weight loss. We identified CAGs, which were promoted by prebiotics and exhibited significant associations with improvements in hepatic steatosis and cholesterol levels. Further research is needed to understand the mechanisms underlying how these prebiotic-enriched guilds of bacteria help in mediating beneficial metabolic responses in the host.

## Declaration of Competing Interest

The authors declare that they have no known competing financial interests or personal relationships that could have appeared to influence the work reported in this paper.
