# Supplementary material for: Active phase prebiotic feeding alters gut microbiota, induces weight-independent alleviation of hepatic steatosis and serum cholesterol in high-fat diet-fed mice
Source: Comput Struct Biotechnol J. 2020 Dec 24;19:448–58. doi: 10.1016/j.csbj.2020.12.011 (PMC7806547; doi:10.1016/j.csbj.2020.12.011)

**Supplementary data**

Fig. S1. Cumulative food consumption (kcal). Data expressed as mean ± SEM and statistical significance assessed by two-way ANOVA, followed by a Tukey *post-hoc* test.

Fig. S2. Consumption of prebiotic prevents loss of intestinal mass and increases total cecum weight in HFD-fed mice. (A) Colon length (n=9/group except HF, n=10) (B) Colon tissue mass (n=10) (C) Cecal tissue mass (n=10) (D) Relative mass of cecum (n=10). Data expressed as mean ± SEM and statistical significance assessed by one-way ANOVA, followed by a Tukey *post-hoc* test. (*p<0.05, **<0.01, ***p<0.001).


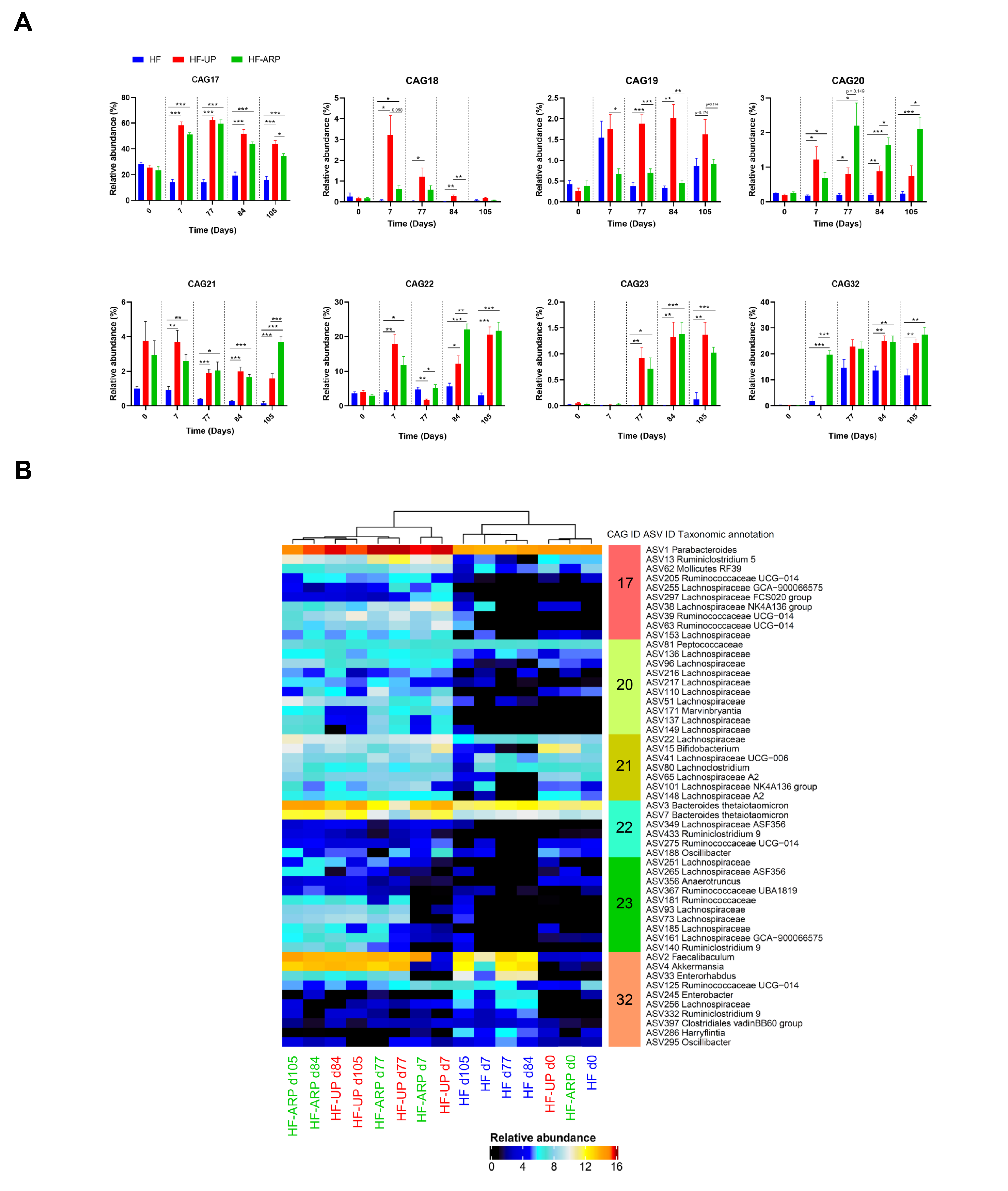


Fig. S3. CAGs promoted by both prebiotic consumption and prebiotic feeding regime. (A) Relative abundance of the CAGs. Data expressed as mean ± SEM and statistical significance assessed by two-way ANOVA, followed by a Tukey *post-hoc* test. (*p<0.05, **<0.01, ***p<0.001). (B) Heatmap of mean relative abundance (log2-transformed) of the CAGs promoted by prebiotic feeding and positively correlated with improvements in hepatic triglyceride and serum cholesterol, showing variation in abundance overtime. Samples (columns) were clustered using Euclidean distance.

­­­­
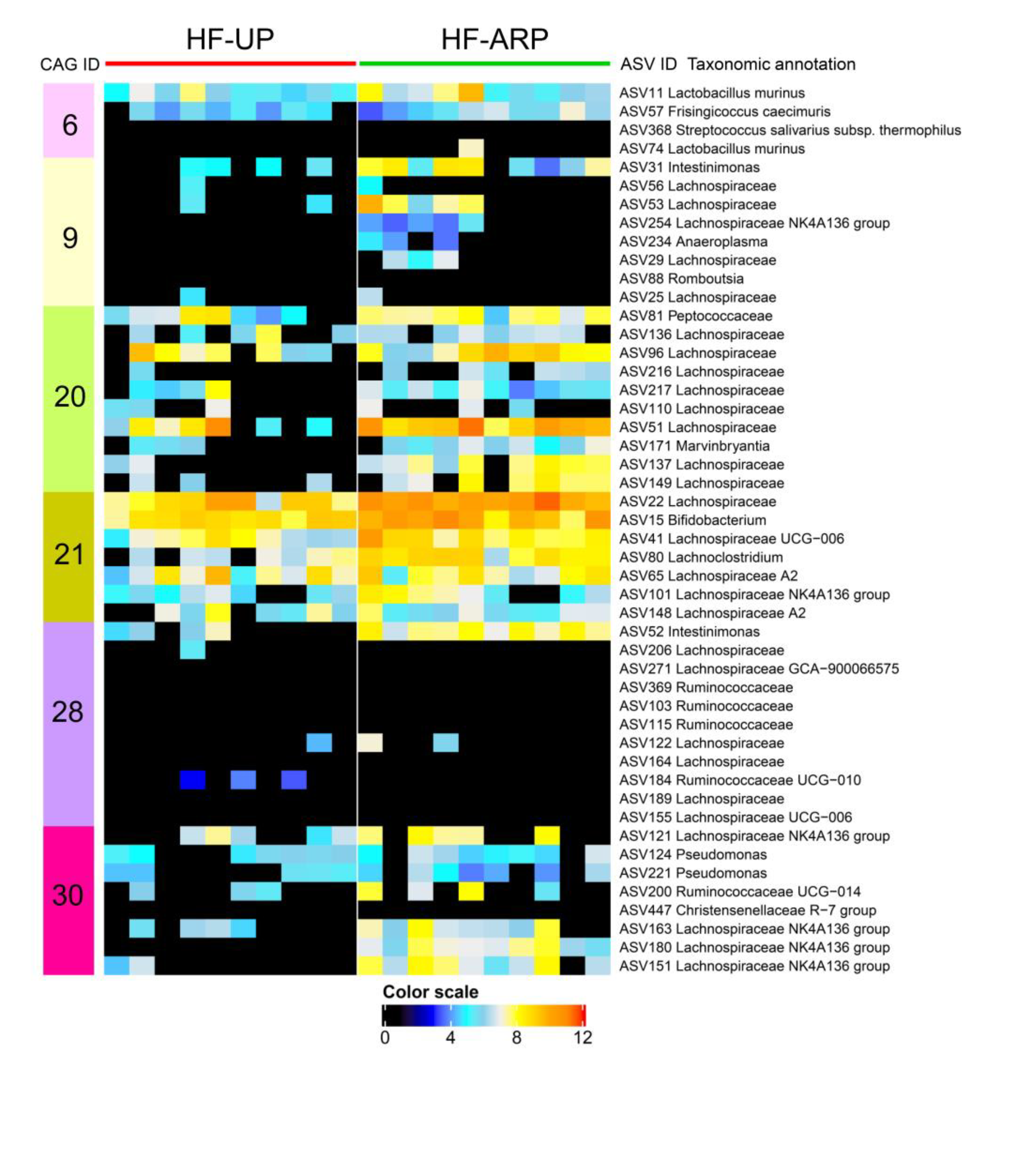


Fig. S4. Alteration in abundance of CAGs induced by prebiotic feeding regime. Heatmap of relative abundance (log2-transformed) of the ASVs listed within the CAGs for HF-UP and HF-ARP for day 105.

Table S1. Composition of high-fat diet (Research Diet 12492)


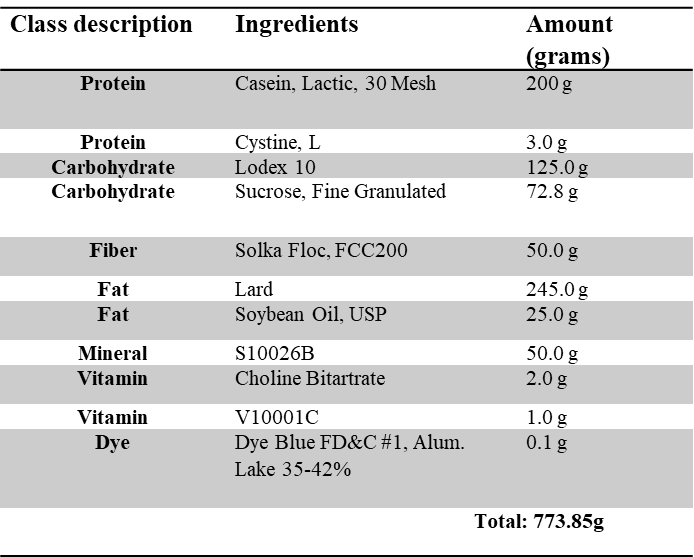


Table S2. Composition of Formula 3


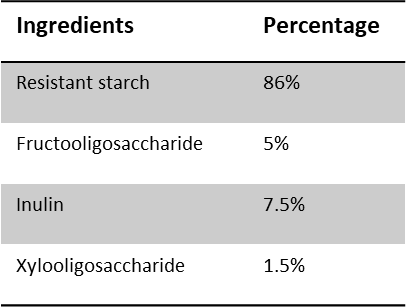


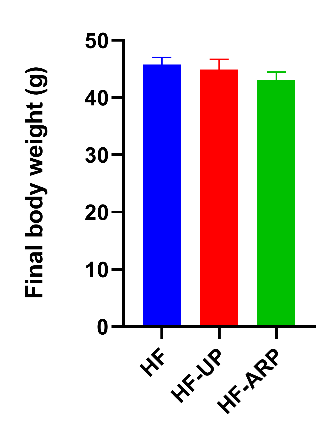


Fig. S5. Final body weight.

Data is expressed as mean ± SEM and statistical significance was by one-way ANOVA followed by a Tukey *post hoc* test for multiple group comparison (n=10).

Fig. S6. Association between SCFA production and hepatic triglyceride.

Pearson correlation coefficient along with p value are indicated on each plot for (A) acetate, (B) propionate, (C) butyrate and (D) total SCFAs.

Fig. S7. PCoA plot based on Bray-Curtis dissimilarity index. Each point represents a sample, the different groups are colored in blue (HF), red (HF-UP) and green (HF-ARP) and the different shapes refer to the timepoints.

Fig. S8. Food and prebiotic intake by cage. (A) Daily food intake per mouse by cage over the study duration (C1= cage1, C2=cage2). Data is expressed as mean ± SD. Daily prebiotic intake per mouse by cage over the study duration for (B) 10% prebiotic intake and (C) 20% prebiotic intake. Data is expressed as mean ± SD.

Table S3. Food and prebiotic intake by cage
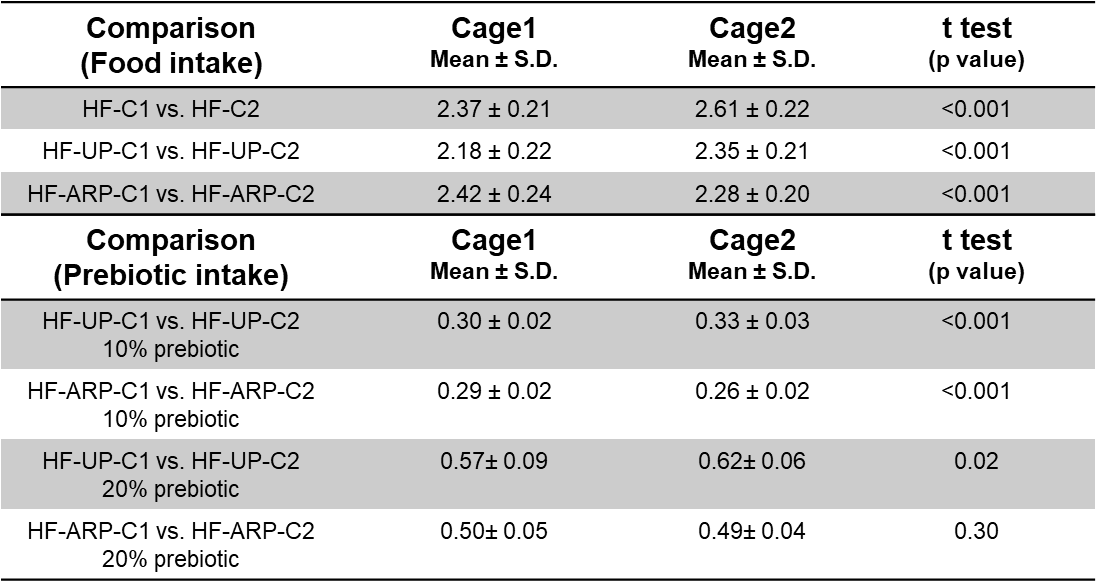


Table S4. Body weight gain by cage
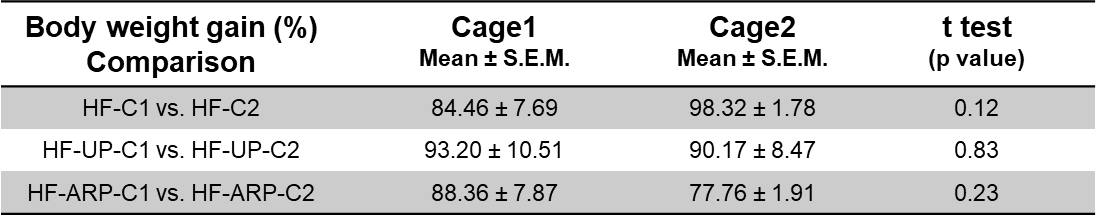

Supplement: Supplementary data 1 [file mmc1.docx]
